# Integrating social and value dimensions into sustainability assessment of lignocellulosic biofuels

**DOI:** 10.1016/j.biombioe.2015.04.022

**Published:** 2015-11

**Authors:** Sujatha Raman, Alison Mohr, Richard Helliwell, Barbara Ribeiro, Orla Shortall, Robert Smith, Kate Millar

**Affiliations:** a Institute for Science & Society (ISS), School of Sociology & Social Policy, University of Nottingham, Nottingham, NG7 2RD, United Kingdom; b Centre for Applied Bioethics (CAB), School of Biosciences and School of Veterinary Medicine and Science, University of Nottingham, United Kingdom

**Keywords:** Lignocellulosic biofuels, Integrated sustainability assessment, Social and value dimensions of technology, Agricultural systems, Responsible innovation

## Abstract

The paper clarifies the social and value dimensions for integrated sustainability assessments of lignocellulosic biofuels. We develop a responsible innovation approach, looking at technology impacts and implementation challenges, assumptions and value conflicts influencing how impacts are identified and assessed, and different visions for future development. We identify three distinct value-based visions. From a techno-economic perspective, lignocellulosic biofuels can contribute to energy security with improved GHG implications and fewer sustainability problems than fossil fuels and first-generation biofuels, especially when biomass is domestically sourced. From socio-economic and cultural-economic perspectives, there are concerns about the capacity to support UK-sourced feedstocks in a global agri-economy, difficulties monitoring large-scale supply chains and their potential for distributing impacts unfairly, and tensions between domestic sourcing and established legacies of farming. To respond to these concerns, we identify the potential for moving away from a one-size-fits-all biofuel/biorefinery model to regionally-tailored bioenergy configurations that might lower large-scale uses of land for meat, reduce monocultures and fossil-energy needs of farming and diversify business models. These configurations could explore ways of reconciling some conflicts between food, fuel and feed (by mixing feed crops with lignocellulosic material for fuel, combining livestock grazing with energy crops, or using crops such as miscanthus to manage land that is no longer arable); different bioenergy applications (with on-farm use of feedstocks for heat and power and for commercial biofuel production); and climate change objectives and pressures on farming. Findings are based on stakeholder interviews, literature synthesis and discussions with an expert advisory group.

## Highlights

• We develop a responsible innovation approach to assessing lignocellulosic biofuel systems.
• We clarify social and value dimensions required for integrated sustainability assessment.
• Insights on farming and agricultural system perspectives are important for sustainability assessment.
• We identify techno-economic, socio-economic and cultural-economic perspectives on lignocellulosic biofuel development.
• Regionally-tailored bioenergy configurations may provide alternatives to large-scale biorefinery models.

## 1 Introduction

Expectations are high for the development and commercialisation of second-generation biofuels as a sustainable way of meeting renewable transport fuel policy targets [8], [13], [49]. Set up in response to sustainability concerns over first-generation biofuels derived from food crops, the UK Gallagher Review [42] called for policies to support biofuels based on non-food feedstocks including perennial crops (miscanthus and short rotation coppice willow) and agricultural residues. In light of controversy over the impacts of first-generation biofuels on food security and indirect land-use change (iLUC), the European Commission proposed amendments to the Renewable Energy and Fuel Quality Directives to cap the share of food-based biofuels in its 2020 renewable transport fuel target and to allow only advanced (non-food) biofuels in the post-2020 framework, though, at the time of writing, the changes are yet to be ratified [12].

Some suggest that second-generation advanced biofuels are unlikely to pose any significant ethical or social challenges (e.g., [6], [35]). However, others recognise the need for more detailed investigation of potential challenges [3], [32], [43]. The Gallagher Review acknowledged that advanced biofuel technologies may have limitations depending on the way they were actually developed and implemented. Established to support research into advanced biofuel options that could overcome the problems of first-generation biofuels, the UK Biotechnological and Biological Sciences Research Council's (BBSRC) Sustainable Bioenergy Centre (BSBEC) therefore included work within one of six projects (Lignocellulosic Conversion to Bioethanol or LACE) on the three pillars of sustainability: environmental, economic and social.

This paper aims to clarify the social and value dimensions of sustainability of lignocellulosic biofuels with reference to the UK. Most published studies of sustainability assessment of biofuels focus on first-generation options, though interest in second-generation options is growing. However, key gaps remain in terms of: first, how the social dimension is understood and second, how well it is integrated into sustainability assessments which mainly focus on life-cycle assessments (LCA) of environmental performance. The paper aims to fill the first gap in order to inform future research intended to address the second. This is done by highlighting the values and assumptions that underpin different visions of how lignocellulosic biofuels production might and should unfold.

In existing literature, social aspects tend to appear in the form of a checklist of generic and biofuel-specific criteria, notably, impacts of biomass feedstock cultivation and processing on food security, water security, employment generation and rural development, visual and noise-level aspects in a community, legal compliance and social acceptance (e.g., [30], [44]). These studies usefully broaden the scope of sustainability assessment of biofuels beyond life cycle assessment. However, the underlying value assumptions in impact assessment and options for developing the technology in different ways are not really addressed. Also, separating social aspects from environmental and economic ones is not always straightforward. Indeed, this is implicit in Markevicius et al [30] as they list the availability and robustness of systems for monitoring an environmental criterion such as greenhouse gas (GHG) accounting in their list of social criteria.

Restricting social assessment to questions of ‘impacts on society’ risks ignoring the social and value judgements involved in choices over which impacts to include and how to assess them [17], [38]. For example, Thornley and Gilbert [56] point out that some environmental impacts (e.g., eutrophication) are location-specific while others (GHG savings) represent gains/losses to the planet as a whole. How then should these different outcomes in what initially appears as a universally shared criterion be valued? Mohr and Raman [32] find that value questions pervade virtually all sustainability criteria including food security, representing both challenges and opportunities for policy making and debate around bioenergy. In addition, there is extensive social research on bioenergy (reviewed in Ref. [39]) considering wider issues that are not typically included in ‘sustainability assessment’. These include aspects related to global trade, land ownership and the potential for social enterprise models that are relevant to biofuel futures (e.g., [63]), but there is currently no place for considering such matters in conventional sustainability assessments. This makes it difficult to identify and evaluate knowledge that is appropriate for biofuel policy making and debate [4]. The paper addresses this gap by distilling the main social and value dimensions that need to be considered in creating lignocellulosic biofuel futures.

## 2 Theoretical framework: responsible innovation in emerging technologies

This research is grounded in insights from a framework of responsible innovation [36], [52] which has been put forward to develop a shared understanding of how to bring societal and value questions to bear on research and innovation. Responsible innovation builds on insights and methods from established work on technology assessment, applied ethics and technology studies [20]. Emerging technologies have been widely assessed in terms of approaches in applied ethics [28], [31] and traditions of technology assessment (TA) such as constructive TA [45] and real-time TA [21]. Responsible innovation research builds on these traditions, but aims to draw more attention to the questions of what innovation *should* do and stimulate reflection on why research is unable to ‘fix’ grand societal challenges despite being framed as a response to them [37], [53]. From this perspective, potential impacts and unintended consequences of emerging technologies are placed in a broader framework of alternative values and visions for their future development.

In the biofuels case, a number of studies have highlighted important considerations from the implementation of first-generation, food-based biofuels including impacts on livelihoods, food and water security, and the local and global environment (e.g., [32], [44] for overviews). But cross-cutting most of these impact indicators are questions of uneven spatial distribution in terms of where biomass has come from, which regions have borne the negative impacts, which ones benefited and what alternative ways might there be of producing biofuels [39]. Understanding the impacts of biofuels requires getting to grips with these value-based matters and disagreements around them [16]. Palmer [38] has shown the problems that arise when a key question such as the impacts of biofuel policies on indirect land-use change has been framed entirely as a factual one, when assessments are wrapped up with value choices over which impacts to measure and how to measure them. Responsible innovation puts such values at the centre of emerging technology assessment with implications for sustainability assessment of biofuels.

A full-fledged application of the responsible innovation framework would require a large-scale programme for anticipating intended and unintended impacts; reflecting within research communities on what is known about these as well as what is not known but may be important; opening up these processes to a wide range of inputs from multiple stakeholders and publics; and building the capacity in the research and innovation process to be responsive to these inputs. This paper provides resources to feed into such future programmes.

Our approach is built on two key insights. First, building on previous insights from constructive and real-time TA, responsible innovation highlights the need for assessment to take note of the wider system in which specific technologies and technological ensembles are developed. This is compatible with the vision of integrating sustainability assessment to include environmental, social and economic dimensions. Since lignocellulosic biofuels may be based on different possible feedstocks, processing technologies and end-uses, we characterise them as a ‘technological system’ rather than a technology. This also allows us to pay closer attention to farm-level and agricultural perspectives than has been typical in bioenergy sustainability assessment.

Second, responsible innovation calls attention to different ways in which an innovation could unfold and the value judgements involved in going down one pathway as opposed to another. This also means that people will assess what is, is not or can be novel about a technological innovation differently [22], [40]. This insight is important for second-generation biofuel futures as what appears to be a technical innovation in relation to oil-based transport could be seen by some as an *extension* of existing socio-economic, institutional and environmental patterns and values. Conversely, a responsible innovation framework opens up the possibility of looking for ways of innovating in social, economic and institutional terms together with technical ones [40]. So, assessing the social and value dimensions of technology can be useful for identifying ways of innovating that have not been widely considered.

## 3 Methods

We draw on empirical research conducted on biofuels including a documentary synthesis of key literature on sustainability assessment and social aspects of biofuels relevant to the UK (notably: [39], [56], [57], [60], [61], [62]); interviews with stakeholders in farming and farm-related intermediary work, bioenergy science, research and industry, policymakers and NGOs; and discussions held with an expert group set up to provide advice on the project. The academic literature cited above is supplemented with studies of specific sustainability dimensions which are cited as appropriate in our summary Tables, and reports on sustainability of second-generation biofuels conducted by major organizations [26], [27]. The expert group consisted of five leading members of the bioenergy research community with expertise in biological sciences, life cycle assessment, sustainability assessment and social sciences.

To apply the responsible innovation framework to sustainability assessment, we develop a model for eliciting key insights from such an extensive information-base. It consists of the following elements.

First, the main impacts of the technological system in question need to be identified. In the case of an emerging technology, these will be *potential* impacts, anticipation of which is strongly likely to be shaped by prior experience – so, second-generation options are likely to be seen in terms of how they compare with first-generation experiences. However, impact identification must remain open to new issues including potentially positive impacts as well as negative ones and important disagreements about these judgements.

Second, since we are assessing a technological system that is not yet widely established, impacts may not actually materialise if it cannot get off the ground. So, potential challenges to establishing/implementing it need to be identified. In the biofuels case, these emerge from looking at the wider agricultural system which is the necessary context for any bioenergy system to develop.

Third, key assumptions and value conflicts that arise in assessing impacts and challenges need to be clarified. Maximising diversity of content in perspectives gathered is therefore more important than getting a representative sample of opinion since we specifically need to understand differences rather than identify the view of a majority. Some underlying assumptions may be based on expectations (what is expected to happen for impacts to unfold) while others may be value-based (judgements on whether particular impacts are desirable or should be avoided). In either case, the aim is to make transparent the assumptions that are the most significant either because they are open to contestation or because they may help identify possible options (once the assumptions are made explicit).

Fourth, taking the above elements together, the analysis needs to draw out different future visions for how the technology might be developed and for what purpose. Each vision will represent different values, but making these explicit can help pave the way for engagement across these differences.

## 4 Social and value dimensions of lignocellulosic biofuel assessment

In this section, we apply the above model for clarifying the social and value dimensions of sustainability assessment of lignocellulosic biofuels. This requires looking in turn at potential impacts (4.1), assumptions and challenges (4.2), value conflicts (4.3) and different future visions (4.4).

### 4.1 Potential impacts of lignocellulosic biofuels

In specifying impacts, we could choose to cast the widest possible net and synthesise an extensive list of criteria from the studies reviewed above. However, although impact characterisation is well-developed in the biofuels literature, even the most exhaustive framework is at risk of leaving out possible elements. For example, it has been suggested that the human health impacts of liquid biofuels have been under-researched [3], [47]; Thornley and Gilbert are one of the few to explicitly consider the impact of biofuels on energy security and Mohr and Raman [32] the implications of ethanol fermentation techniques for antimicrobial resistance. Equally, expanding analysis to cover all possible impacts – including ones that are generic in nature and could arise from virtually any project – may result in inadvertently downplaying the most crucial aspects. Mohr and Raman [32] and Ribeiro [43] take the route of focussing on the most significant criteria relevant to second-generation biofuels, but since their analysis is based on existing knowledge of first-generation biofuels, it is not clear how new criteria – i.e., not previously considered in the first-generation case – can be identified through this approach. Overall, there are tradeoffs between exhaustiveness, significance and making room for novel issues in impact analysis.

We therefore propose a streamlined approach with three core elements – essential resources for human use, environmental impacts, and amenities – that can be used to classify and investigate already known categories of impact as well as encourage attention to inquiring about new possibilities raised by changes to the technological system in question. These core elements cover the most cited issues about biofuels, i.e., impact on food security and greenhouse gas balances, but also allow us to broaden the analysis to other environmental impacts [3] and to ask how biofuels might impact on energy security. This last aspect is usually assumed rather than investigated. Framing the elements this way also enables us to consider potentially *positive* impacts which are often ignored in assessments of sustainability ‘challenges’ and for *whom* impacts might be positive or negative. This latter question is crucial as much of the controversy around first-generation biofuels is related to concerns about the uneven North/South distribution of negative impacts versus benefits [39], [62].

We do not make assumptions at the start as to where lignocellulosic biomass or the end-product, cellulosic ethanol, is expected to come from. Rather we assume that lignocellulosic biomass will be sourced and processed within a globally integrated biofuel network [33] that characterises first-generation biofuels at present. This is because prominent policy documents such as the UK Bioenergy Strategy [8] assume some role for imports of biomass and biofuels in meeting 2020 or 2050 decarbonisation targets. However, we move on to consider a theoretical UK-based biofuel system based on domestically sourced feedstocks, which has been proposed as a solution to the North/South problems of a globally distributed system.

#### 4.1.1 Resource impacts

The impact of biofuel development on essential resources – food, water and energy – can be explored under this heading. Framed in terms of access to resources and impacts on access, we can also begin to ask whose access is affected and how (i.e., improvements versus threats to access). The inclusion of energy access in this framework helps draw attention to the first of the three purposes cited for biofuel development – energy security, climate change mitigation, rural development – and ask if this has been met and for whose benefit.

Again, it is worth reiterating that since we are talking about lignocellulosic biofuels which are yet to be deployed on a significant scale, the purpose of the analysis is to clarify if specific impact criteria are *potentially* relevant and, if so, why and in what context. Later, we will consider responses to some of the concerns about negative impacts. Table 1 summarises potential impacts associated with sourcing and processing/conversion of the three most-discussed feedstocks: perennial crops, crop residues and forestry (woody, fibrous) residues. There is emerging interest in other feedstocks such as the biomass component of municipal solid waste (e.g., see Ref. [27]) but these are beyond the scope of this paper.

Table 1, Table 2 aim to capture and summarise the most commonly encountered concerns (expectations of negative impacts indicated by −) and arguments on behalf of lignocellulosic biofuel development (expectations of positive impacts marked in the Table as +), as well as judgements of impacts being negligible to non-existent (represented as ‘nil’). In some cases, a ‘nil’ judgement may be good if it means that negative side-effects are minimal or manageable so as to be minimal; in other cases, it represents a problem if the side-effects being sought are positive (e.g., if impact on energy security is nil, this would be construed a problem). It is important to recognise that + and – judgements rely on particular *conditions* and the aim is to make these explicit. The final column summarises how impacts on each resource are likely to be distributed in a production system that is globally distributed, i.e., where either lignocellulosic biomass or the end-product is traded across borders. The judgements in Table 1, Table 2 are based on our analysis of documents, stakeholder interviews and advisory group discussions as a whole to identify the most important themes and assumptions. The method is broadly similar to recent work also oriented towards synthesizing impact assessments results, in this case, for bioenergy heat options for a university-sized facility [60].

A key point to note from Table 1 is the potential for lignocellulosic biofuels to reproduce some of the controversies around the first-generation, but this depends on how the technology is actually developed and implemented. The purpose of this analysis therefore is to make explicit expectations of intended benefits followed by some key concerns about the uneven distribution of negative impacts that might arise.

Expectations of benefits from lignocellulosic biofuels for food security and energy security are based on the assumption that lignocellulose will/can (eventually) replace current use of food crops for biofuels without itself clashing with food priorities, and reduce use of petroleum in an age where demand is forecast to increase due to population growth and development. However, these positive expectations are tempered by concerns about land availability if energy crops were to encroach on land for food production; arguments that biomass is far less energy-dense than fossil fuels and therefore cannot reproduce ‘Western’ lifestyles; and constraints posed by a 10% blending limit under current vehicle designs.

Concerns about potentially negative impacts are informed by the assumption that, in practice, lignocellulosic biofuel development in a global economy can clash with food and energy security with incentives favouring sourcing of feedstocks from poorer Southern countries for use in the richer North. Monitoring sustainability impacts (land-use change, use of residues for fuel) under these conditions is harder [62]. This is partially contested by the argument that lignocellulose is too bulky to transport and that countries are more likely to import the finished product, i.e., liquid fuel. But if this were the case, there is still a key concern: that poorer populations from the region of production will experience only negative impacts (e.g., location-specific environmental risks) and none of the positive ones (improved energy security). Also, the original concern can be seen as a warning – should the techno-economics of transporting lignocellulose become more favourable in future, current incentives may change.

Similarly, the difference made by technological improvements is interpreted differently – for advocates, these can ‘fix’ potentially negative impacts, but for others, there is a danger that improving crop traits or the efficiency of lignocellulosic conversion processes will be accompanied by a rebound effect where overall energy demand is driven up rather than down [1]. This may well happen without improvements to the energy security of poorer countries – in other words, it cannot be assumed that increasing the overall level of fuel produced will automatically meet the needs of energy-poor populations.

#### 4.1.2 Environmental impacts

Here, in addition to the wider database (mentioned in Sections 3, 4.1), we draw on Borrion et al's [3] literature review of life cycle assessments (LCA) of lignocellulosic bioethanol in particular. Since LCA studies specifically look across the full life cycle, we do not distinguish in the summary (Table 2) between the biomass component and the processing/conversion component – the contributions to each of these to overall environmental outcomes are typically reported in individual studies, but we do not have space to delve into this.

Table 2 shows that the environmental impacts of lignocellulosic biofuels are uncertain because much depends on the assumptions made in LCAs on scenarios under which crops would be grown or residues gathered as well as scientific difficulties in measuring multiple impacts under different possible scenarios. However, recognising these limitations can alert us to social and value questions that remain important in considering environmental implications. First, the distribution of impacts will again be uneven as we saw with access to resources – in a global production system, some regions will bear the costs (should they arise) of production and not necessarily the benefits. Second, advocates or critics may choose a particular LCA that supports their view but this ignores the fact highlighted by Ref. [3] that findings depend on system boundaries, functional unit of analysis, quality of data and allocation methods which vary considerably across studies as the judgements made by different authors vary. Third, these authors also point out that some potential impacts are ignored in published studies or under-researched – for example, the impact of enzymes and catalysts used in biofuel production. Antibiotic use in bioethanol fermentation and its impact on antibiotic resistance is another example of such a category [32]. Fourth, as lignocellulosic ethanol is not widely commercialised, published LCAs of real-world operations are few. Using LCA to predict possible impacts (consequential LCA) is challenging as actual impacts will depend on the choices made in practice (e.g., on land-use for crop cultivation) which are likely to differ from those assumed in the model (e.g., if the model assumes that worst-case iLUC scenarios will be avoided).

#### 4.1.3 Amenity impacts

Under the heading of amenities, we can include other criteria that commonly figure in sustainability assessments of biofuels, notably, negative impacts (−) around noise levels, traffic from transportation of bulky biomass to refineries, and visual landscape impacts of energy crop cultivation (miscanthus, in particular). By definition, these are local impacts experienced at particular sites of cultivation, transport and refining. Some research in the UK on public opinion suggests that landscape aesthetics of crops were of less concern than significant changes from the wider infrastructure of bioenergy plants including heavier traffic; people also wanted to know how the local community would benefit [10]. These, however, could be interpreted as characteristic of any new industrial development rather than specific to lignocellulose.

#### 4.1.4 Economic impacts

This aspect is perhaps the least developed in the sustainability assessment literature. Criteria cited include impacts on job creation, on the local economy and contributions to national and global economies. Of these, impacts at a local community or regional level are likely to have the most concrete meaning in the first instance – by contrast, the impact of one industry is harder to isolate in national or global figures unless it becomes significantly large as in the case of Brazilian sugarcane ethanol. Whether lignocellulosic biofuels will develop on such a scale remains an open question, but we will pick up on this when we explore future visions. For now, it should be noted that economic impacts can, as with any industrial project, also be negative. This can happen either if investments by biofuel producers fail in the marketplace, or, ironically, when they do succeed but by creating incentives for greater economies of scale which leads to amalgamation of smaller farms and the hollowing-out of rural communities [64]. In a global production system, these impacts will be likewise distributed globally – the farmers affected will include those in Southern countries with energy crop investments by multinational companies.

### 4.2 Options to reduce negative impacts: assumptions and challenges

In terms of most of the key potential impacts identified above – on resource access, environment, amenities and economic aspects – fundamental differences between lignocellulosic and first-generation biofuels are few. In anticipation of some concerns about negative impacts, advocates of lignocelluosic biofuels propose the following options:

• Source lignocellulose domestically (e.g., a UK biofuel production system relying on UK-grown feedstocks)
• Improve crop traits so as to reduce some negative impacts and increase positive ones (for example, higher yield, less water usage, improved water quality)
• Develop a biorefinery model that would yield high value-added by-products as well as fuel and hence make the process more efficient in energy and economic terms

Of these, attempts to improve crop traits and biorefinery models are part of ongoing research looking to shape future possibilities and we therefore examine them under future visions (4.4). For now, we focus on unpacking the assumptions, and potential challenges and opportunities around proposals to source lignocellulose within the UK for UK biofuel purposes. The vision of a domestic UK biofuel system is based on a.) cultivating perennial crops on ‘marginal land’ to avoid or reduce competition with arable land [59] and b.) the use of residues from food crops, notably, wheat and barley straw. In principle, the vision for use of marginal land partly addresses findings such as those reported by Glithero et al [18] who found in a survey of 244 English farms that over 80% of arable farmers would *not* consider switching to dedicated energy crops.

Domestic sourcing would not in itself address all the potential negative impacts considered in the previous section (e.g., impact of removing straw from the soil). But, as we saw, most of these are not clear-cut as studies report different findings. In Table 3, we focus instead on the ability to create a domestic UK-based system in the way that has been proposed, looking specifically at some key assumptions embedded in the vision for use of marginal land for perennial crops. Shortall [48] distinguishes between two meanings of marginal land, one referring to land that some believe *should* be used for perennial crops as it is unsuited to food production (we call this ‘Marginal land I’) while the other captures the notion of land that is more likely to be used because it is currently economically marginal (we call this ‘Marginal land II’). In the next column, we draw on interviews with livestock farmers in North West England, data from which was presented and discussed at one of our expert advisory meetings, to consider practical challenges of implementing the vision. For residues, we focus on the case of wheat straw, drawing on a recent farm survey by Glithero et al [19]; interviews with agronomists (who play a key role in advising farmers) and wheat farmers (to see if they would be willing to supply straw for biofuel) and related analysis in our overall research project.

Table 3 highlights a number of challenges to the vision of sourcing lignocellulosic feedstocks within the UK – expectations of surplus biomass or available (marginal) land are countered by judgements on the ground by farmers. It also shows that farmers are not the only group of actors influencing what happens as they face multiple pressures; and that farm-level decisions are not solely shaped by market/profit incentives. Farming/rural cultures and the legacy of existing norms, practices and infrastructures (including relationships and advice from other key intermediaries) also matter. These present barriers, but may also open up new ways of thinking about the case for perennial crops which we will return to in Section 4.5.

#### 4.2.1 Fundamental challenge posed by trade rules

In addition to the above, there is a broader challenge to the vision of UK-sourced feedstocks for biofuels that has not yet been widely recognised. Subsidies for creating domestic biomass markets in place of foreign-sourced biomass or biofuel may be deemed to have a global trade-distorting effect prohibited by World Trade Organization (WTO) rules. This has been raised by industry stakeholders in interviews; it is also explored in a few studies offering contradictory conclusions [11], [23], [55]. This issue requires further attention.

### 4.3 Value conflicts in systems assessment of lignocellulosic biofuels

So far, we have considered the potential impacts of lignocellulosic biofuels and conditions shaping the ability to develop them within the UK (an objective stated partly in anticipation of some negative impacts). We have emphasised the open-endedness of judgements of impacts and challenges/opportunities for two reasons:

• *Conditionality*: Judgements of impacts/challenges involve assumptions about the context or conditions under which technologies are introduced [65].
• *Valuation*: Judgements of impacts/challenges are linked to value-based assumptions even where impacts are quantifiable [56]

The responsible innovation framework set out in Section 2 calls for a systems approach to technology assessment. This requires paying attention to issues and questions raised in relation to lignocellulosic biofuels that relate to the broader socio-technical system in which they would be introduced. Many of the conditions and value-judgements raised in Table 1, Table 2, Table 3 reflect the perspective of those looking at this wider system. A systems view also means asking questions about what is included/excluded within environmental assessments of the technology, the supply chain ‘system’ that is being modelled and its boundaries and assumptions. Biofuel appraisal must therefore elucidate the different value-frameworks that inform these judgements [16], [17].

#### 4.3.1 Value priorities

Broadly three different value-frameworks can be identified across the assessments summarised in Table 1, Table 2, Table 3. Each prioritises a different core value and each has its own yardstick or reference point for assessment (Table 4).

##### 4.3.1.1 Techno-economic proficiency

Expectations of positive impacts are shaped by the assumption that lignocellulosic biofuels represent a novel and effective solution to the problems of first-generation biofuels and the fossil-fuel economy, and that any environmental side-effects or negative social impacts can be managed by a combination of improved technology and sustainability monitoring mechanisms. The yardstick for assessment is the performance of first-generation biofuels and fossil fuels.

##### 4.3.1.2 Socio-economic justice

Warnings of negative impacts are informed by the view that the lignocellulosic biofuels solution (as currently developed) is not novel enough because it is likely to fit within and reinforce the values of an unequal global economy. The yardstick for assessment here is the global socio-economic system and whether lignocellulosic biofuels can/will distribute environmental and social impacts more fairly than at present. Trust in the capacity of global sustainability monitoring schemes to address these issues is low under conditions skewed towards the interests of large landowners and companies by comparison with smallholders and local communities [58], [62] and it is not yet clear how this will change with lignocellulosic feedstocks.

##### 4.3.1.3 Cultural economic preservation

Expectations of challenges (as opposed to either positive or negative impacts) are grounded in recognition of the value of existing practices that lignocellulosic biofuel advocates seek to transform. The yardstick for assessment here is the legacy of farming knowledge, norms, skills and cultures, in this case, in the UK. Lignocellulosic biofuel development as currently proposed is seen to be at odds with the value of this legacy and the multiple pressures faced by UK farmers at present. It should be noted that with the same yardstick, the valuation may turn out differently in other countries, i.e., where lignocellulosic biofuels are seen as compatible with farming values and agricultural systems.

#### 4.3.2 Value-based questions

The above value-frameworks are ideal-types, so, it is possible for assessments to be framed by more than one of them or integrated in new ways. In order to do this, we need to clarify the most significant value questions.

##### 4.3.2.1 Distribution of impacts

As we have seen, there are key value questions in considering how to assess and manage the impacts of new technologies. Which impacts are included? Who decides and how? How is the uneven distribution of impacts dealt with especially if some communities bear the costs of biofuel development and others the benefits? These questions are certainly not unique to biofuels, let alone lignocellulosic systems, but they are crucial to its future. They do not figure within a techno-economic framework, but are central to a socio-economic one. From a cultural-economic framework, the focus tends to be on farming communities rather than the system as a whole.

##### 4.3.2.2 Valuation of land and land uses

How should land be valued, managed and used? This question is emerging as a key one around the biofuels debate. Importantly, it begins to challenge assumptions of a food-versus-fuel divide as the primary criterion of assessment [15], [29], [34], [39]. It does so by opening up for scrutiny two key issues about the sustainability of food production – the large-scale cultivation of grain for animal feed, and the energy footprint of farming – with implications for bioenergy. Rather than taking food production to be the sole and primary purpose of agricultural land-use, some bioenergy experts (e.g., [50]) and environmental campaigners (e.g., [5]) have developed scenarios in which some land is released from existing uses for animal feed (and hence, food production) and made available for bioenergy. A related environmentalist vision, *Two Energy Futures*, arising from a UK campaign against tar sands also includes a place for lignocellulosic biofuels.^2^

Interestingly, the case for increasing biofuel production by reducing meat consumption has been made from *both* a techno-economic and a socio-economic perspective as represented by environmental campaigners. From a techno-economic view, it is inefficient to use large tracts of land for grain to feed animals bred for human consumption, so it makes better sense to turn over some of this land for bioenergy. From a socio-economic view, further (social) conditions are spelled out as might be expected: the cultivation of energy crops without creation of large monoculture plantations remote from place of consumption; and cultivation by small-scale farmers and cooperative/social enterprises who have more control over what is grown and how, and over access to benefits.

By contrast to both techno-economic and socio-economic values, a cultural-economic framework would imply more caution on the more-biofuel/less-meat vision. Livestock farming would be seen as part of a cultural tradition with its own skills and norms that cannot be destroyed lightly. However, there may be opportunities for exploring the evolution of less industrialised livestock farming models that are amenable to mixing with energy crops. In this respect, there may be commonality with the socio-economic critique of monocultures.

##### 4.3.2.3 Valuation of biomass

What are the best uses of biomass given multiple, competing priorities? This question has been raised in policy documents on bioenergy [9] and related commentary [7] through the notion of a ‘hierarchy of best use’. One view is that biomass should be reserved for applications where it can be used most efficiently which means that combined heat and power (CHP) and heat would be prioritised over liquid transport fuel [7]. By contrast, a rival view is that the use of lignocellulose for transport fuel should be given some priority as there are few current alternatives to liquid fuel for decarbonising aviation, shipping and long-haul vehicles [25], [27]. Rather than seeing hybrid vehicles as a competing alternative, some also suggest that liquid biofuels could significantly contribute to diffusion of hybrids (e.g., [25]). A third view is that a ‘step-change’ in economic efficiency of lignocellulosic conversion is possible if liquid fuel production were to be combined with the production of value-added products (bio-based chemicals, materials) in a biorefinery [51], [66]. All three perspectives share a techno-economic framework, defining best use in terms of the criterion of efficiency. By contrast, from a socio-economic framework, other criteria become relevant for defining best use: who chooses, who benefits, how are impacts distributed? From a cultural-economic framework, on-farm use to meet the farm's own energy needs becomes part of the mix of criteria.

##### 4.3.2.4 Valuation of nature

How should nature be valued? Does nature have intrinsic value apart from providing ‘services’ for human use? These questions have not played a significant role in the biofuels debate so far, but they represent an established tradition in environmental philosophy which may become more significant as biomass and the land from which it is sourced are seen as ever more valuable ‘resources’ to fulfil more functions than ever before.

### 4.4 Future visions for UK-based lignocellulosic biofuels

In this section, we consider the implications of the different value-based perspectives and questions outlined above for lignocellulosic biofuel futures. Table 4 summarises the three main perspectives we identified, the key question arising from each and the vision that is articulated in response.

With techno-economic proficiency as the core value, the future vision for lignocellulosic biofuels is centred on improving biomass traits to fulfil energy needs in a biorefinery process yielding high-value products in addition to fuel. Production would be self-sufficient in energy terms with co-products used to power the conversion process.

With socio-economic justice as the core value, lignocellulosic biofuels might feature within an agricultural system that departs from the present in terms of its smaller scale and level of regional control. In principle, this would allow development of such fuels in different regions across the world (so, not just the UK) where appropriate biomass resources can be made available, but local communities would have more control over the system and its impacts.

With preservation of the cultural economy of (UK) farming as core value, energy crops emerge only by building on opportunities around current farming skills and practices rather than from a rapid transformation. For example, one opportunity that has been recently put forward is the option of growing miscanthus on arable land made unprofitable (‘marginal’) by bedrock and blackgrass weed. The weed is difficult to control, but unlike arable crops, miscanthus can still flourish in such an environment [14].

Note that prioritising techno-economic value involves focussing on the technology (lignocellulosic biofuels) or technological system (biorefining) while socio-economic and cultural-economic value prioritisation draws attention to the system in which these are expected to take root. However, as these are ideal-types, there is potential for learning and engagement across different visions in charting the future. A reframed model might start with the following elements which emerged from our advisory group discussions. These cannot resolve once and for all the value conflicts identified, but could provide a way for navigating them and opening up a more widely informed discussion.

#### 4.4.1 Regionally specific

Rather than looking for a one-size-fits-all model of developing lignocellulosic biofuels (e.g., as implied in the biorefinery vision), it may be better to look for regionally specific models that are appropriate to local agricultural needs and resources and to explore their societal robustness in conjunction with members of the public in the region. For example, flood management is an urgent requirement in some areas (where perennial energy crops might be beneficial) while improved crop traits would be most useful in areas where food crops are challenging to grow (or might become more challenging with climate change).

#### 4.4.2 Social enterprise

The lignocellulosic-biorefinery model is based on the assumption of greater efficiency from economies of scale which in turn requires large commercial enterprises that are able to manage globally distributed operations. More attention to ownership of land, resources and operations could open up alternative smaller-scale partnership or social enterprise models which are gaining more attention around renewable energy [63]. Similar models are being explored to bring in forms of smallholder participation in Southern countries in global bioenergy networks that benefit local people [54].

#### 4.4.3 Doing agriculture better

While grand visions of transforming the agricultural system are difficult to implement wholesale, a regionally-centred model might allow more concrete engagement with existing agricultural practices and appropriate avenues for innovation to meet multiple needs (food, fuel including for farming, waste management, biodiversity). This could help rethink the food-versus-fuel-versus-feed divide to consider ways of integrating these in practice – for example, experiments to mix SRC willow with livestock grazing, or crops providing all three elements of food, feed and fuel. It would open up different ways of conceiving of multipurpose agriculture beyond large biorefineries alone.

## 5 Conclusions

A case for lignocellulosic biofuels in the UK has been made from a techno-economic perspective. According to this perspective, the technology could be developed to contribute to energy security with greenhouse gas benefits (i.e., lower emissions) and few or none of the problematic sustainability impacts of first-generation biofuels and fossil fuels. This vision appears especially promising when based on a domestically sourced feedstock chain which might be more trusted to manage potentially negative side-effects on the environment and promote positive ones, although the precise nature of impacts on biodiversity, soil quality, air and water pollution and water availability is contested due to different assumptions made in different life cycle assessment studies. In this vision, food security, the foremost concern in debates on first-generation biofuels, is expected to be largely unaffected with the use of crop residues and perennial crops grown on marginal land.

In this paper, we have applied a responsible innovation perspective to assessing the sustainability of the above vision for lignocellulosic biofuels, focussing specifically on social and value dimensions. This required attending to different assumptions about the conditions under which the technology would be implemented and other value-based perspectives against which potential impacts are judged. From a socio-economic perspective, lignocellulosic biofuels are seen as problematic mainly because they are expected to emerge within the existing global agricultural economy. Given that biomass is a globally traded commodity and subsidies for domestic supply chains may fall afoul of World Trade Organization (WTO) rules, there is some validity to this concern. Due to embedded inequalities between multinational entities, regional elites and the poor, it is also harder to monitor and manage sustainability impacts within globally distributed networks. From a cultural-economic perspective, building domestic supply chains will be challenging given tensions with the legacy of farming skills, norms and practices, and the multiple pressures faced by farmers. The extent to which there is sufficient ‘marginal land’ in the UK on which to sustainably cultivate energy crops for a domestic supply chain is also in question.

These differences in perspective leave us with two key questions for future work. Neither socio-economic nor cultural-economic perspectives on biofuels engage with the question of how second-generation biofuels compare with fossil fuels. From a socio-economic perspective, the comparison is not usually made – possibly because of the judgement that both fuel systems arise from the same global economic system which itself needs changing. From a cultural-economic perspective, it is likely that agricultural stakeholders have their own ideas on how farming should adapt to sustainability threats to fossil-fuel-based systems (e.g., see Ref. [41]). Efforts to promote second-generation biofuels will need to engage with these independent visions. In either case, scepticism about the capacity of biofuels to fulfil expectations given the blend wall in current vehicle design and lower energy density of biomass will need to be addressed.

Second, in light of these different value-based perspectives, what might be some promising alternative options for the future of biofuels? Here, there is an opportunity to work at regionally specific levels across techno-economic, socio-economic and cultural-economic perspectives and explore ways of combining environmental aspirations for lowering meat consumption, reducing reliance on monocultures, easing pressures on farming with diversified business models including smaller-scale and social enterprise options, and generating fossil-fuel alternatives. This could mean reconciling some aspects of first-generation biofuels with the second-generation, for example, with some element of ‘feed’ crops used together with lignocellulosic co-products. It would also mean making visible different applications and uses of bioenergy including on-farm uses for reducing the energy footprint of agriculture through a broader range of conversion technologies as suggested in a recent Royal Agricultural Society of England report [41]. Rather than trying to create public acceptance for a single technologically-defined vision centred on large-scale lignocellulosic biofuels and biorefineries, this strategy for responsible innovation calls for opening up different policy options for configuring different bioenergy applications in technological, environmental, social and institutional terms.

In conclusion, it should be noted that sustainability assessment cannot resolve the complexities and conflicts value conflicts identified here, but can help make them more transparent for policy-making and debate.

## Figures and Tables

**Table 1:** Potential impacts of a globally distributed lignocellulosic biofuel production system on access to resources.

Resource	Perennial crops	Crop residues	Forestry residues	Processing & conversion	Distribution of impacts
Food	(+) If this helps replace current use of food crops for fuel (−) If crops were grown on land of value for food	No direct competition with food, but: (+) if this helps replace use of food crops for fuel (−) If residues are part of animal feed (e.g., straw)	No direct competition; indirect impacts not assessed	No direct impact, so (nil)	(−) impacts globally distributed, disproportionately affecting lower-income populations relying on the market for accessing food; (−) local or regional impacts where biomass is sourced by displacing people from common property land
Water	Could be (−) in current conditions in the case of miscanthus which requires high water inputs. Can be (−) if crops are grown in areas requiring irrigation. Technological improvements may compensate [29] impact if crops are grown at smaller scale But (+) impact also from water retention due to year-round cover from perennial crops [26]	Not widely studied. But residue cover reduces evaporation of water from soil, conserving moisture. (−) impact of residue removal on water therefore cited as a risk [2], but potentially manageable with careful choice of sites. Where residues become commodities in their own right, how water use is allocated between the crop and co-products may be at stake. Overall: (nil/–)	Similar issues about residue cover conserving moisture are relevant [46]. Overall: (nil/–)	Based on current processes, lignocellulosic conversion is more water-intensive than first-generation due to added conversion steps [26]; hence, (−). Provided improvements occur (e.g, [26] highlight modern plants improving recycling practices), impacts could be (nil) in future Overall: (nil/–)	Likely to be local, affecting specific areas where biomass is sourced
Energy	(+) for fuel-users if it helps meet transport energy needs as expected. But this is constrained by lower energy-density of biomass and the ‘blend’ wall (in vehicles as currently designed) (−) If the biomass competes with other bioenergy options	(+) for fuel-users if it helps meet transport energy needs as expected. But this is constrained by lower energy-density of biomass and the ‘blend’ wall (in vehicles as currently designed) (−) If the biomass competes with use for other bioenergy options	(−) if residues are sourced from areas where they fulfil energy needs of forest-dwellers	(−) in current conditions where energy inputs for processing & conversion are high. Overturning (−) impact possible through technological improvements	Likely to be distributed globally as well as local & regional effects – e.g., (−) impacts where residues have local fuel use in subsistence farming [64]. Contribution to energy security of poorer countries may therefore be (nil)

**Table 2:** Potential environmental impacts of a globally distributed lignocellulosic biofuel production system.

Environmental outcome	Perennial crops (inc processing & conversion)	Crop residues (inc processing & conversion)	Forestry residues (inc processing & conversion)	Distribution of impacts
Greenhouse gas emissions	Most studies report (+) impact, i.e., GHG savings compared to fossil fuels [26] but actual impact on the ground depends on choices about land-use	Most studies report (+) impact, i.e., GHG savings compared to fossil fuels [26]; But including (−) impact of residue removal on soil carbon would change the assessment [27]	Studies reporting (+) impact, i.e., GHG savings challenged by others reporting high potential for (−) impact, i.e., increase in GHG emissions from iLUC (due to diversion of forestry residues from current uses in furniture, paper/pulp industries) or from total increase in use of forestry resources [27], [46]. Impact of residue removal on soil carbon also cited as (−) [27]	Impact on national carbon savings targets. Physical impacts of GHG changes will be felt on a global level.
Biodiversity	Some evidence of (+) impact on biodiversity [24]. Depends on indicators used for biodiversity, and what perennial crops are compared to: arable crops, grassland or leaving the land fallow. Or if compared with fossil fuel extraction, high (+) impact [26]	IEA (201) suggests it depends on whether these are ‘primary’ or ‘secondary’ residues. (−) impact for primary residues which are likely to be left as cover or ploughed back into soil with benefits for microflora. (+) impact for secondary residues which would be treated as waste. (−) impact may be ameliorated depending on how much residue is removed vs left behind	Similar concern about (−) impact due to role of forest residue cover in enhancing soil biodiversity. Here Schulze et al [46] suggest even thinning of forest cover (as opposed to large-scale deforestation) can be harmful	Locally specific impacts experienced in sites of biomass sourcing
Water, Soil & Air quality	Contrary results of + and – reported across studies. Review suggests more research is needed especially on under-researched aspects (e.g., impact of enzymes, catalysts used in conversion).	Contrary results of (+) and (−) across LCAs. Depends on allocation of impacts between crop and co-product. Frequently cited concern of (−) impact on soil structure and nutrient balance from removing residues (vs ploughing back to soil). Difficulties with scientific measurement of impacts on soil [26]	(−) impact on soil quality and structure from removing residues cited as concern Difficulties with scientific measurement of impact on soil quality [26]	Local impacts experienced in and around sites of biomass sourcing and conversion

**Table 3:** Assumptions, challenges and opportunities around a UK-based lignocellulosic biofuel system.

Source of UK system	Assumptions implicit in proposals	Challenges to assumptions (& potential opportunities)
Marginal land I for perennial crops (land that should be used as it is unsuitable or less suitable for food production)	There is sufficient land of this type available in the UK Production for lignocellulosic biofuel is technically and economically possibly on such land, despite lower quality It is possible in a market economy to restrict energy crops to such land, avoiding higher-quality land where yield (and profits) would be higher	In interviews, farmers agreed in principle to the idea that perennial energy crops should be grown on marginal land. But, for the most part, they did not consider their own land to be of marginal quality & in this sense, available for perennial crops. Legacy, pride in their work, skills developed over time with existing infrastructure and machinery, and their role in the wider rural community were crucial to farming identity. Farmers are not just profit-maximisers; they face multiple pressures due to changing sustainability demands, Common Agricultural Policy reforms, power of large supermarket chains as main buyers, etc. Changing to perennial crops may be an added challenge in this context.
Marginal land II for perennial crops (land that is currently economically marginal that is more *likely* to be used)	If some food production is displaced on this type of land (identified in Ref. [59] as grades 3 and 4), technological improvements (higher yields) will compensate Grassland (also identified as economically marginal in Ref. [59] is assumed to be suitable for conversion to cropland – but this is contested by others highlighting the release of soil carbon emissions	Farmers overwhelmingly took food production to be the moral purpose of farming, though their view of technological improvements compensating for displaced food is unclear Livestock farmers took their grassland to be of ‘prime quality’ in the sense of prime for grazing for their animals Where they judged some parts of their land to be economically marginal, multiple competing uses were foreseen
Crop residues (case of wheat straw)	Surplus of cereal straw estimated to exist in the UK with potential for bioenergy uses	Two-thirds of farmers in recent survey indicated they would be willing to supply wheat straw for bioenergy [19], though this would include different bioenergy applications, not only liquid fuel In interviews, farmers indicated they have little control over the end-use for baled straw as these decisions are taken by straw merchants while straw merchants indicated preference to preserve their existing customer base in non-fuel sectors There are tensions between bioenergy aspirations for using straw and the UK Code of good Agricultural Practice which recommends incorporating straw into soil, a message reinforced by agronomists

**Table 4:** Different value priorities and Future visions for lignocellulosic biofuels.

Core value	Yardstick for comparison	Key question	Future vision
Techno-economic proficiency	First-generation biofuels & fossil fuels	Can lignocellulosic biofuels be economically sustainable with lower environmental costs than these established energy technologies?	Transforming Biomass in Biorefineries
Socio-economic justice	Global Agri-Economy	Can systems for producing lignocellulosic biofuels distribute environmental, social and economic impacts more fairly than present systems?	Transforming Agricultural Systems
Cultural-economic preservation	Regional farming practices, skills & knowledge (UK)	Can energy crops for lignocellulosic biofuels work with established legacies of (UK) farming and the pressures it faces?	Evolving Agricultural Practices

## Footnotes

2 http://www.twoenergyfutures.org/accessed 29.8.2014.
